# Concentration of Tobacco Advertisements at SNAP and WIC Stores, Philadelphia, Pennsylvania, 2012

**DOI:** 10.5888/pcd12.140133

**Published:** 2015-02-05

**Authors:** Amy Hillier, Mariana Chilton, Qian-Wei Zhao, Dorota Szymkowiak, Ryan Coffman, Giridhar Mallya

**Affiliations:** Author Affiliations: Mariana Chilton, Drexel University School of Public Health, Philadelphia, Pennsylvania; Qian-Wei Zhao, University of Pennsylvania School of Social Policy and Practice, Philadelphia, Pennsylvania; Dorota Szymkowiak, Ryan Coffman, Giridhar Mallya, Philadelphia Department of Public Health, Philadelphia, Pennsylvania.

## Abstract

**Introduction:**

Tobacco advertising is widespread in urban areas with racial/ethnic minority and low-income households that participate in nutrition assistance programs. Tobacco sales and advertising are linked to smoking behavior, which may complicate matters for low-income families struggling with disparate health risks relating to nutrition and chronic disease. We investigated the relationship between the amount and type of tobacco advertisements on tobacco outlets and the outlet type and location.

**Methods:**

By using field visits and online images, we inspected all licensed tobacco retail outlets in Philadelphia (N = 4,639). Point pattern analyses were used to identify significant clustering of tobacco outlets and outlets with exterior tobacco advertisements. Logistic regression was used to analyze the relationship between the outlet’s acceptance of Supplemental Nutrition Assistance Program (SNAP) and Special Supplemental Nutrition Program for Women, Infants, and Children (WIC) and the presence of tobacco advertisements.

**Results:**

Tobacco outlets with exterior tobacco advertisements were significantly clustered in several high-poverty areas. Controlling for racial/ethnic and income composition and land use, SNAP and WIC vendors were significantly more likely to have exterior (SNAP odds ratio [OR], 2.11; WIC OR, 1.59) and interior (SNAP OR, 3.43; WIC OR, 1.69) tobacco advertisements than other types of tobacco outlets.

**Conclusion:**

Tobacco advertising is widespread at retail outlets, particularly in low-income and racial/ethnic minority neighborhoods. Policy makers may be able to mitigate the effects of this disparate exposure through tobacco retail licensing, local sign control rules, and SNAP and WIC authorization.

## Introduction

Tobacco products are heavily marketed in retail spaces in urban communities. Combined with ready availability and low prices, advertising can increase consumption of unhealthy products and contribute to poor health. Previous research from multiple cities showed that tobacco outlets and tobacco advertising are concentrated in low-income and racial/ethnic minority areas and near child-serving institutions (1–5). Studies also showed an association between smoking rates and the number of outlets and advertisements (6,7). Outdoor and point-of-sale tobacco advertising increase smoking initiation among adolescents, undermine smokers’ quit attempts, and promote relapse among former smokers (7–16).

Smoking rates in the United States are strongly associated with income, with lower-income populations having higher rates of smoking (17). In Philadelphia, 34% of adults living below the federal poverty guidelines, 37% of participants in the Supplemental Nutrition Assistance Program (SNAP), and 23.1% of participants in the Special Supplemental Nutrition Program for Women, Infants, and Children (WIC) are smokers, compared with 20% of nonpoor adults (27). Nationally, smokers earning less than $30,000 per year spend 14% of their income on cigarettes, exacerbating already challenging financial circumstances for themselves and their families (28). Over the life course, low socioeconomic status is associated with higher rates of smoking initiation and regularization and lower rates of quitting (27). Among Latinos, smokers are 30% to 50% more likely to be food insecure than nonsmokers, controlling for sex, age, and poverty status (17).

Because the largest 4 tobacco companies agreed in the Master Settlement not to advertise tobacco products on billboards, advertisements for tobacco products at tobacco retail outlets are the most prevalent form of tobacco advertising. These accessory or “on-premise” advertisements are regulated differently from nonaccessory advertisements such as billboards.

For this study, we used a survey and digital photographs to analyze the location of tobacco outlets in Philadelphia and the type and amount of tobacco advertisements on their exterior and interior. In addition to clustering of tobacco outlets relative to each other, proximity to schools, and the income and racial/ethnic characteristics of neighborhoods, we considered the association between retail outlet type — including whether outlets were authorized to accept SNAP and WIC — and different types of tobacco advertisements.

## Methods

### Sample

Tobacco retailers are readily identifiable through state and local licensing. Retailers in Philadelphia are required to have state and city licenses to sell tobacco products. The Philadelphia Department of Public Health (PDPH) provided our team with an initial list of 4,513 retailers in the Philadelphia Tobacco Retailer Database (PTRD) in October 2011. PDPH provided an updated list, yielding a combined total of 4,639 retailers, in February 2012.

We developed a survey based on existing instruments and discussions with PDPH staff. The survey was programmed using Pendragon 5.1 software (Pendragon Software Corporation) for HP iPAQ personal digital assistants (Hewlett-Packard Co). Student and community assessors participated in training to conduct the assessments and take digital photographs of the exterior of the outlets; this training included a presentation, detailed protocol, and practice field work. Assessors were instructed to take photographs from the opposite side of the street and take multiple photographs if needed to capture the complete façade. The project manager accompanied all staff on field work at least once and compared independently collected assessments by each assessor to address any discrepancies in data collection.

Between November 2011 and July 2012, assessors visited as many of the retailers as possible (N = 3,970). They coded outlets based on whether they were operational or permanently closed and whether they sold tobacco products.

All retail outlets not visited because of time and resource constraints or that were closed during the initial visits were reviewed by using Google Street View (https://www.google.com/maps/views/streetview?gl=us) in November 2013 with images dated between July 2011 and September 2012. Outlets that were coded as closed during the initial visit and appeared closed in the Google images were coded as permanently closed. All other outlets were coded as operating tobacco retail outlets unless no outlet could be identified. Outlets were then geocoded with ArcGIS 10.1 (Esri) using the address in the PTRD.

### Tobacco advertisements

Assessors provided store staff with an information card about the study translated into Spanish, Chinese, Vietnamese, Cambodian, Korean, and Hindi. Only if assessors could not see pricing on tobacco products did they ask for assistance. Assessors recorded 1) all tobacco products (regular cigarettes, menthol cigarettes, cigars, chewing tobacco, snuff or dipping tobacco, or wraps [flavored rolling papers]) advertised on the exterior; 2) the products advertised in the 5 largest interior tobacco advertisements, based on their visual inspection of size; and 3) the total number of interior tobacco advertisements. Assessors also indicated whether tobacco advertisements were immediately above, below, or next to products targeted to children, including potato chips, candy, toys, and sugary drinks. The project manager and 1 community assessor reviewed all of the digital photographs to determine that they met specifications and then coded them for the total number and location of tobacco advertisements.

### Tobacco retail outlet type

Outlets were categorized as a chain convenience store, local corner store (less than 2,000 square ft, single cash register), grocery store (primarily sold food, larger than corner store but smaller than full-service supermarket), supermarket, dollar store, gas station, newsstand, chain pharmacy, local pharmacy, laundromat, check-cashing store, smoke shop, bar, beer distributor, beer-to-go, restaurant/take-out, deli, or other. Each outlet was also identified as being authorized or not to accept SNAP or WIC. A list of authorized SNAP vendors was obtained from the US Department of Agriculture’s SNAP retail locator website (18), and a list of authorized WIC vendors was obtained from the Pennsylvania Department of Health WIC Program. Because SNAP and WIC use different identification systems from the PTRD and because the outlet name reported may have differed, SNAP and WIC outlets could not be matched to the PTRD using only identification numbers or outlet names. Instead, SNAP and WIC outlet addresses were geocoded using ArcGIS 10.1 and matched to tobacco outlets on the basis of having exact *x* and *y* coordinates. SNAP and WIC status was then confirmed by reviewing outlet names, addresses, and images from Google Street View.

### Characteristics of retail outlet locations

The type of street and the zoning classification for each outlet were determined by using the spatial join feature in ArcGIS 10.1. Street type was defined by using the 2013 Philadelphia street centerline definitions of arterial (major or minor; characterized by lights at most intersections and speeds of 35 mph or greater). Zoning was identified as residential or not residential.

A geocoded list of all public, private, and charter schools with any grades kindergarten through 12 was obtained from the City of Philadelphia. Tobacco outlets were coded based on the presence of a school within 500 ft (Euclidean distance).

The locations of tobacco outlets were joined to census tract–level estimates from the American Community Survey (ACS) for 2008 through 2012 on racial composition (percentage black/African American) and poverty (percentage of individuals living below federal poverty guidelines). These data were later dichotomized to identify tracts with greater than 50% black population, 30% to 50% below poverty guidelines, and greater than 50% below poverty guidelines. Data on smoking rates at the zip code level came from the 2012 Southeastern Pennsylvania Household Health Survey (http://www.chdbdata.org/householdsurvey.html).

### Statistical analysis

Bivariate and multivariate logistic regression models were used to identify characteristics of outlets and their location associated with exterior advertisements, interior advertisements, and interior advertisements near products targeted to children. Only variables found to have a significant bivariate association were entered, simultaneously, into the multivariate models.

Ripley’s local K-function was used to test for significant clustering of tobacco outlets. Ripley’s local K-function compares the distribution of events (tobacco outlets) to randomly generated point patterns to test the null hypothesis of complete spatial randomness (19). Because the null hypothesis may be unrealistic (ie, tobacco outlets would not be expected in parks or rivers), the randomly generated patterns are weighted based on a “backcloth” (total population in 2010 by census block group). Clustering was tested by using search radii of 2,000 and 2,500 ft. Ripley’s local cross–K-function was used to test for significant clustering of tobacco outlets with exterior tobacco advertisements relative to all tobacco outlets. The location of tobacco outlets was compared with 500 randomly generated simulations to determine a clustering *P* value. The cross–K-function uses a marked point process and can be used to compare subgroups of a single population to the distribution of all points in the region (20). The location of tobacco outlets with exterior tobacco advertisements relative to the location of all tobacco outlets was compared with 500 randomly generated point patterns using search radii of 2,000 and 2,500 ft.

All study protocols were approved by the University of Pennsylvania Institutional Review Board (IRB) and the PDPH IRB.

## Results

Of the 4,639 outlets included in the February 2012 version of PTRD, assessors completed surveys at 2,805 (60.5%). Of the remaining outlets, 10.1% were visited but they were not selling tobacco, 7.8% were visited but no retailer was found at the location, 5.0% were permanently closed, 2.0% were duplicate listings, 2.0% were viewed online and were closed, and 12.5% were viewed online and appeared to be selling tobacco.

Many of the retail outlets with tobacco licenses were also authorized to accept SNAP: 45.9% of the tobacco outlets were authorized to accept SNAP, and 81.9% of SNAP stores were licensed to sell tobacco. Nearly all WIC-authorized stores (97.7%) were also authorized to accept SNAP benefits, but only 1 in 3 SNAP-authorized stores (35.1%) was also authorized to accept WIC. Most WIC-authorized stores (72.3%) were licensed to sell tobacco.

Of the 3,356 tobacco outlets identified as open and selling tobacco products, 50.3% had at least 1 exterior tobacco advertisement. Of outlets with exterior tobacco advertisements, 18.4% were within 500 ft of a school. Of the 2,805 outlets where surveys were completed, 69.1% had at least 1 interior tobacco advertisement and 19.7% had tobacco advertisements near products targeted to children (Table 1).

**Table 1:** Distribution of Tobacco Outlets (N = 3,356) by Retail Store Type and Presence of Tobacco Advertisements, Philadelphia, Pennsylvania, 2012

Type of Outlet	Total Outlets, N (% of Total)	Outlets With Exterior Advertisements, n (%)	Outlets With Interior Advertisements, n (%)	Outlets With Advertisements Near Children’s Products, n (%)
**Store type**				
Chain convenience	116 (3.5)	81 (69.8)	100 (86.2)	14 (12.1)
Corner store	1,236 (36.8)	847 (68.5)	831 (67.2)	378 (30.6)
Grocery store	249 (7.4)	169 (67.9)	179 (71.9)	57 (22.9)
Supermarket	79 (2.4)	12 (15.2)	54 (68.4)	1 (1.3)
Dollar store	38 (1.1)	26 (68.4)	18 (47.4)	1 (2.6)
Gas station	225 (6.7)	184 (81.8)	176 (78.2)	32 (14.2)
Newsstand	131 (3.9)	66 (50.4)	67 (51.1)	12 (9.2)
Chain pharmacy	137 (4.1)	3 (2.2)	123 (89.8)	9 (6.6)
Local pharmacy	45 (1.3)	12 (26.7)	23 (51.1)	3 (8.6)
Laundromat	42 (1.3)	10 (23.8)	10 (23.8)	3 (6.7)
Check-cashing store	68 (2.0)	31 (45.6)	40 (58.9)	2 (2.9)
Smoke shop	36 (1.1)	24 (66.7)	25 (69.4)	3 (8.3)
Bar	59 (1.8)	3 (5.1)	5 (8.5)	0
Beer distributor	99 (2.9)	50 (50.5)	70 (70.7)	5 (5.1)
Beer-to-go	128 (3.8)	81 (63.3)	91 (71.1)	10 (7.8)
Restaurant/take-out	525 (15.6)	44 (8.4)	72 (13.7)	19 (3.6)
Deli	30 (0.9)	8 (26.7)	0	0
Other	113 (3.4)	40 (35.4)	53 (46.9)	3 (2.7)
**Accepts SNAP**	1,542 (45.9)	964 (62.5)	1,102 (71.5)	389 (25.2)
**Accepts WIC**	542 (16.2)	380 (70.1)	391 (72.1)	179 (33.0)
**School located within 500 ft**	656 (19.5)	311 (47.4)	361 (55.0)	129 (19.7)

Abbreviations: SNAP, Supplemental Nutrition Assistance Program; WIC, Special Supplemental Nutrition Program for Women, Infants, and Children.

In the bivariate logistic regression models, many of the independent variables were significant across the dependent variables, including SNAP and WIC status, downtown location (Center City), moderate poverty (30%–50%), and large black population (Table 2). Gas stations (odds ratio [OR], 4.83; 95% confidence interval [CI], 3.42–6.82) were the most likely to have exterior tobacco advertisements. Chain pharmacies (OR, 58.75; 95% CI, 8.20–421.12) were the most likely to have interior tobacco advertisements. Corner stores (OR, 4.79; 95% CI, 3.92–5.86) were the most likely to have tobacco advertisements near products targeted to children.

**Table 2:** Bivariate Logistic Regression Models of Tobacco Outlet Retail Store Type and Characteristics of Location by Type of Tobacco Advertisements, Philadelphia, Pennsylvania, 2012

Characteristic	Exterior Advertisements, OR (95% CI)	Interior Advertisements, OR (95% CI)	Near Children’s Products, OR (95% CI)
**Store type**			
Chain convenience	2.34 (1.56–3.50)	9.39 (3.81–23.14)	0.62 (0.35–1.09)
Corner store	3.27 (2.82–3.79)	1.85 (1.56–2.20)	4.79 (3.92–5.86)
Grocery store	2.20 (1.67–2.89)	1.60 (1.16–2.20)	1.37 (1.00–1.88)
Supermarket	0.17 (0.09–0.32)	1.63 (0.91–2.90)	0.06 (0.01–0.42)
Dollar store	2.15 (1.08–4.27)	1.15 (0.48–2.77)	0.17 (0.02–1.25)
Gas station	4.83 (3.42–6.82)	10.74 (5.26–21.91)	0.85 (0.57–1.26)
Newsstand	1.00 (0.70–1.42)	0.80 (0.53–1.21)	0.52 (0.28–0.96)
Chain pharmacy	0.02 (0.01–0.06)	58.75 (8.20–421.12)	0.31 (0.16–0.61)
Local pharmacy	0.35 (0.18–0.69)	0.86 (0.42–1.73)	0.38 (0.12–1.24)
Laundromat	0.30 (0.15–0.62)	0.15 (0.07–0.31)	0.34 (0.10–1.10)
Check-cashing store	0.82 (0.51–1.33)	1.05 (0.59–1.87)	0.15 (0.04–0.60)
Smoke shop	2.38 (1.13–4.99)	2.82 (0.98–8.13)	NA
Bar	0.87 (0.69–1.11)	1.44 (1.05–1.98)	NA
Beer distributor	1.05 (0.70–1.57)	3.58 (1.78–7.19)	NA
Beer-to-go	1.73 (1.20–2.49)	2.20 (1.33–3.63)	NA
Restaurant/take-out	0.07 (0.05–0.09)	0.06 (0.05–0.08)	0.17 (0.11–0.27)
Deli	0.37 (0.16–0.84)	NA	NA
Other	0.54 (0.36–0.79)	0.61 (0.40–0.94)	0.13 (0.04–0.43)
Accepts SNAP	2.50 (2.18–2.88)	3.91 (3.27–4.67)	3.38 (2.76–4.13)
Accepts WIC	2.72 (2.23–3.32)	3.23 (2.44–4.27)	3.45 (2.77–4.29)
**Location characteristic**			
Arterial street	0.80 (0.69–0.94)	0.98 (0.81–1.17)	0.51 (0.42–0.62)
Center City	0.40 (0.30–0.54)	0.43 (0.32–0.58)	0.19 (0.10–0.37)
Residential	1.09 (0.91–1.30)	0.84 (0.69–1.02)	1.19 (0.95–1.49)
Poverty^a^ >50%	0.92 (0.75–1.11)	0.53 (0.42–0.65)	0.75 (0.56–0.99)
Poverty^a^ 30%–50%	1.41 (1.23–1.63)	0.95 (0.81–1.12)	1.33 (1.10–1.61)
Black population >50%	1.48 (1.26–1.70)	0.90 (0.77–1.06)	1.74 (1.44–2.10)
School located within 500 ft	0.98 (0.85–1.13)	0.79 (0.65–0.96)	1.30 (1.04–1.63)

Abbreviations: CI, confidence interval; NA, not applicable; OR, odds ratio; SNAP, Supplemental Nutrition Assistance Program; WIC, Special Supplemental Nutrition Program for Women, Infants, and Children.

a Proportion of the population living below federal poverty guidelines.

In multivariate analyses, SNAP and WIC status were significantly and positively correlated with all 3 dependent variables, with the strongest relationship between SNAP stores and interior tobacco advertisements (OR, 3.43; 95% CI, 2.80–4.20) and the weakest between WIC status and exterior tobacco advertisements (OR, 1.59; 95% CI, 1.27–2.00) (Table 3). Being in a tract where 30% to 50% of the population lives in poverty was a significant and positive predictor of exterior advertisements (OR, 1.19; 95% CI, 1.00–1.41) but only up to 50%, at which point the direction switched and the relationship was no longer significant. Outlets in Center City were significantly less likely to have all 3 types of advertisements, with ORs ranging from 0.32 to 0.63. Similarly, outlets within 500 ft of schools were less likely to have exterior and interior advertisements (not significant) but were significantly more likely to have tobacco advertisements near products targeted to children (OR, 1.40; 95% CI, 1.10–1.79). Being in a tract with 50% or more black residents increased the odds of exterior advertisements or advertisements near products targeted to children but decreased the odds of having interior advertisements.

**Table 3:** Multivariate Logistic Regression Results for Tobacco Advertisement Type by Tobacco Outlet Retail Store Type and Characteristics of Outlet Location, Philadelphia, Pennsylvania, 2012

Characteristic	Exterior Advertisements, OR (95% CI)	Interior Advertisements, OR (95% CI)	Near Children’s Products, OR (95% CI)
Accepts SNAP	2.11 (1.80–2.47)	3.43 (2.80–4.20)	2.29 (1.81–2.90)
Accepts WIC	1.59 (1.27–2.00)	1.69 (1.23–2.32)	1.90 (1.48–2.44)
School located within 500 ft	0.91 (0.76–1.08)	0.84 (0.68–1.04)	1.40 (1.10–1.79)
Black >50%	1.26 (1.08–1.48)	0.74 (0.61–0.91)	1.42 (1.13–1.77)
Poverty^a^ 30%–50%	1.19 (1.00–1.41)	0.74 (0.60–0.92)	1.08 (0.85–1.36)
Poverty^a^ >50%	0.95 (0.77–1.18)	0.35 (0.27–0.45)	0.65 (0.46–0.90)
Center City	0.63 (0.45–0.87)	0.38 (0.27–0.54)	0.32 (0.15–0.66)
Arterial street	1.00 (0.85–1.18)	1.27 (1.04–1.56)	0.65 (0.52–0.80)
Constant included in the model	0.60	1.80	0.16

Abbreviations: CI: confidence interval; OR, odds ratio; SNAP, Supplemental Nutrition Assistance Program; WIC, Special Supplemental Nutrition Program for Women, Infants, and Children.

a Proportion of the population living below federal poverty guidelines.

The Ripley’s local K-function showed significant clustering of tobacco outlets relative to population distribution and outlets with outdoor tobacco advertisements relative to the distribution of tobacco outlets. The Figure shows significant clustering of tobacco outlets based on a search radius of 2,500 ft in parts of North, South, and West Philadelphia and Center City. The Figure also shows significant clustering of exterior tobacco advertisements relative to the distribution of tobacco outlets in subsections of some of those areas, in particular in Kensington and South Philadelphia. Nearly all of the areas showing significant clustering — with the exception of commercial areas with low residential populations in Center City and South and Southwest Philadelphia — had poverty rates above 25%. Many of those areas also demonstrated above-average smoking rates.

**Figure:**
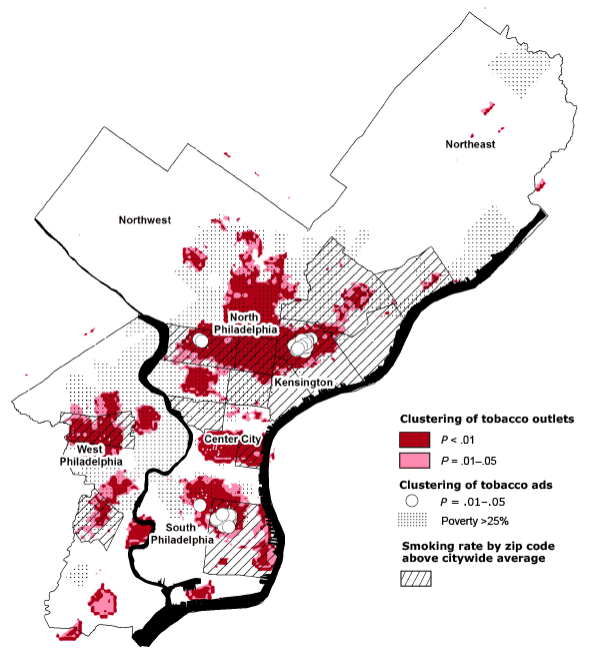
Clustering of tobacco outlets and tobacco advertisements relative to high-poverty areas and zip codes with above-average smoking rates, Philadelphia, Pennsylvania, 2012. Ripley’s local K-function with search radii of 2,500 ft was used to test for significant clustering of outlets.

## Discussion

The most striking finding from this study was that SNAP and WIC status was significantly associated with greater likelihood of exterior advertisements, interior advertisements, and interior tobacco advertisements near products targeted to children, even when controlling for neighborhood poverty and racial composition. When we included a categorical variable for food stores (including chain convenience store, corner store, gas station, grocery store, supermarket, and deli) in the multivariate model, the WIC and SNAP status were no longer significant predictors of exterior advertisements, but WIC and SNAP status were still significant predictors of interior advertisements and advertisements near products targeted to children. Previous research (21) and our own interviews with tobacco outlet staff indicated that representatives from tobacco manufacturers commonly dictate the number and location of exterior and interior tobacco advertisements as part of contracts they establish that impact the prices at which outlets can sell tobacco products. The significance of SNAP and WIC status in our models raises the possibility that tobacco distributors are deliberately targeting SNAP and WIC stores. Alternatively, these outlets may have greater tobacco sales and thus attract the attention of tobacco distributors. Regardless, the effect is that, by virtue of redeeming their federal nutrition benefits, SNAP and WIC participants are likely to be exposed to tobacco sales and advertising.

We also found that controlling for store type, tobacco outlets in areas with large populations of blacks were more likely to have tobacco advertisements placed near products targeted toward children. This may reflect a disproportionate amount of low-cost single-serving sweet and salty products that draw children into corner stores in these communities (22,23). Including racial composition as a continuous variable or dichotomizing the percentage of black population based on a higher concentration did not yield significant results, suggesting that even a slight majority of black residents has the same effect.

Consistent with previous research (2,24), our study found that areas with moderate poverty (30%–50% below federal poverty guidelines) were more likely to have exterior tobacco advertisements and advertisements near products targeted to children. But we also found that outlets in areas with extreme poverty (>50%) were less likely to have all 3 types of advertisements. This could be due to the mix of store types in these neighborhoods. Twenty percent of tobacco retailers in these areas are restaurants/take-outs — a higher proportion than in other areas and a store type that is less likely to have tobacco advertisements. The lower likelihood of advertisements could also be due to lower tobacco sales in very poor communities, despite the fact that smoking rates are high.

Generally, this study demonstrated that tobacco outlets and advertisements for tobacco products are clustered in lower-income neighborhoods in Philadelphia that also have higher-than-average smoking rates. Such environments likely contribute to and perpetuate racial/ethnic and socioeconomic disparities in smoking and, possibly, food insecurity. Policy makers should consider these findings when making decisions about tobacco retail licensing, local sign control (advertising) rules, and SNAP and WIC authorization.

Among the limitations of this study was our inability to complete field visits at all tobacco outlets and the nonrandom nature of the sample not visited. We did not assess inter-rater reliability other than to have the project manager accompany all assessors on their initial visits. Geocoding tobacco outlets and SNAP and WIC outlets separately, then matching them based on *x* and *y* coordinates, may have prevented us from identifying all of the tobacco outlets that accept SNAP or WIC. This study was cross-sectional; the amount and content of advertisements change regularly. Differences in state-level tobacco licensing and municipal sign control regulations also limit the generalizability of our findings beyond Philadelphia. Among the strengths of this study was the commitment to verifying tobacco outlet locations and status and observing interior tobacco advertising practices as well as the use of multivariate regression and point pattern analysis.

Certain policy approaches could mitigate the disparate exposure to tobacco advertising in stores serving low-income populations and in predominantly black/African American neighborhoods. First, federal and state agencies responsible for setting rules on SNAP and WIC participation should consider incentive-based or restrictive approaches or both, as allowable by law, to reduce advertising for unhealthful products and increase advertising for more healthful products in retail spaces. The US Department of Agriculture has asked for comments on how to revise its guidelines for determining retailer eligibility to participate in the SNAP program (25). Both the intended and unintended consequences of such strategies should be considered, including whether they might exacerbate food insecurity. Second, some jurisdictions have limited the number or density of tobacco outlets through zoning rules (26), while others, like Boston and San Francisco, have banned tobacco sales in pharmacies. Third, content-neutral restrictions on retail signage, limiting the amount of window or wall space or both that can be used for advertising, had been implemented in cities such as Los Angeles, California; Austin, Texas; and, recently, Philadelphia. Because such a large portion of retail advertising in corner stores is for tobacco products, sugary drinks, junk foods, and alcohol, even content-neutral restrictions may decrease the amount of advertising for unhealthful products (2). Fourth, although First Amendment hurdles remain, the 2009 federal Tobacco Control Act gives states and localities the ability to restrict the time, place, and manner of advertising. Moreover, the Food and Drug Administration is still considering whether to prohibit exterior tobacco advertisements within 1,000 ft of schools. (Our results from Philadelphia show that 60% of tobacco outlets, and 31% of outlets with exterior tobacco advertisements, are within 1,000 ft of a school.) A final strategy would be to encourage retailers to voluntarily agree not to sell tobacco, following the models of Target and CVS (29), or via exclusionary zones already promoted by the Outdoor Advertising Association of America that prohibit stationary advertisements for products illegal for sale to minors within 500 ft of schools, playgrounds, and places of worship.

Further research is needed on how best to mitigate the health effects of tobacco advertising in communities. State and localities can further these efforts by making information about tobacco outlets available to researchers and requiring outlets to report WIC and SNAP status when applying for tobacco retail permits. The US Department of Agriculture should invest in research to better understand the role tobacco sales play in SNAP and WIC stores.
